# Ruthenium(II) complexes with 6-methyl-2-thiouracil selectively reduce cell proliferation, cause DNA double-strand break and trigger caspase-mediated apoptosis through JNK/p38 pathways in human acute promyelocytic leukemia cells

**DOI:** 10.1038/s41598-019-47914-x

**Published:** 2019-08-07

**Authors:** Larissa M. Bomfim, Fênix A. de Araujo, Rosane B. Dias, Caroline B. S. Sales, Clarissa A. Gurgel Rocha, Rodrigo S. Correa, Milena B. P. Soares, Alzir A. Batista, Daniel P. Bezerra

**Affiliations:** 1 Gonçalo Moniz Institute, Oswaldo Cruz Foundation (IGM-FIOCRUZ/BA), Salvador, Bahia Brazil; 2 Department of Biomorphology, Institute of Health Sciences, Federal University of Bahia, Salvador, Bahia 40110-902 Brazil; 3 Department of Chemistry, Federal University of Ouro Preto, Ouro Preto, Minas Gerais, 35400-000 Brazil; 4 Department of Chemistry, Federal University of São Carlos, São Carlos, São Paulo, 13561-901 Brazil

**Keywords:** Pharmacology, Haematological cancer

## Abstract

Ruthenium(II) complexes with 6-methyl-2-thiouracil *cis*-[Ru(6m2tu)_2_(PPh_3_)_2_] (**1**) and [Ru(6m2tu)_2_(dppb)] (**2**) (where PPh_3 = _triphenylphosphine; dppb = 1,4-bis(diphenylphosphino)butane; and 6m2tu = 6-methyl-2-thiouracil) are potent cytotoxic agents and able to bind DNA. The aim of this study was to evaluate *in vitro* cellular underlying mechanism and *in vivo* effectiveness of these ruthenium(II) complexes in human acute promyelocytic leukemia HL-60 cells. Both complexes displayed potent and selective cytotoxicity in myeloid leukemia cell lines, and were detected into HL-60 cells. Reduction of the cell proliferation and augmented phosphatidylserine externalization, caspase-3, -8 and -9 activation and loss of mitochondrial transmembrane potential were observed in HL-60 cells treated with both complexes. Cotreatment with Z-VAD(OMe)-FMK, a pan-caspase inhibitor, reduced Ru(II) complexes-induced apoptosis. In addition, both metal complexes induced phosphorylation of histone H2AX (S139), JNK2 (T183/Y185) and p38α (T180/Y182), and cotreatment with JNK/SAPK and p38 MAPK inhibitors reduced complexes-induced apoptosis, indicating DNA double-strand break and activation of caspase-mediated apoptosis through JNK/p38 pathways. Complex **1** also reduced HL-60 cell growth in xenograft model. Overall, the outcome indicated the ruthenium(II) complexes with 6-methyl-2-thiouracil as a novel promising antileukemic drug candidates.

## Introduction

Acute promyelocytic leukemia (APL), a subtype of acute myeloid leukemia (AML), is a fatal disease characterized by a reciprocal translocation between chromosomes 15 and 17. From 2006–2012, the overall five-year survival rate for AML was only 27%, and although APL being responsive to treatment containing all-trans retinoic acid and arsenic trioxide, early death, associated with characteristic bleeding diathesis, has emerged as an important cause of treatment failure^1–3^. Therefore, development of new therapeutic approaches that result in better clinical outcome and survival for patients with AML are needed.

Platinum-based antineoplastic agents represent 50% of all antineoplastic regimens. Nevertheless, application of these complexes are restricted by their side effects that include severe nephrotoxicity, neurotoxicity and ototoxicity^4^. Ruthenium-based metallodrugs have been emerged as a novel potential antineoplastic class with less side effect than platinum-based complexes. Therefore, many ruthenium complexes have been synthesized and assessed for their antineoplastic potential in both preclinical and clinical stages, with encouraging outcomes^5–16^.

**Figure 1:**
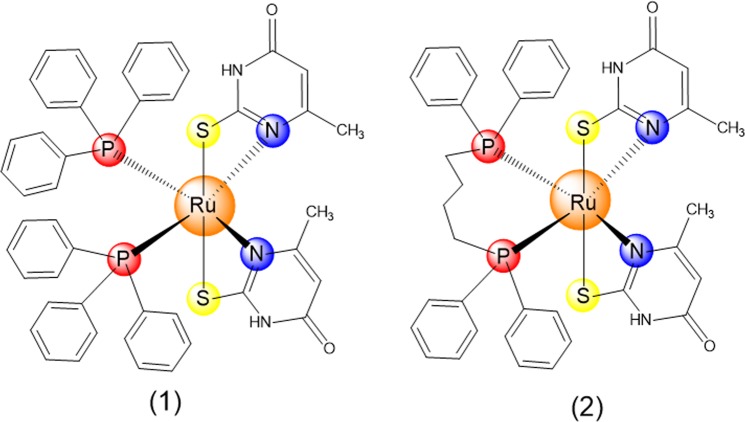
Chemical structure of ruthenium(II) complexes **1** and **2**.

The antineoplastic potential of the metal-based complexes dependent on the nature of the ligands, and metallodrugs complexes containing nucleobases and their derivatives have been previously explored. These include ruthenium(II) complexes with thymine and 5-fluorouracil^9,11^; palladium(II) complexes with 2-thiouracil ligands^17^; gold(I) complexes with 2-thiouracil, 2-thiocytosine and 2-mercaptopyridine^18^; and copper(I) complexes with 2-thiouracil, 6-methyl-2-thiouacil and 4-methyl-2-mercaptopyrimidine^19^. Recently, we synthesized four ruthenium(II) complexes with thiouracil derivatives using [RuCl_2_(PPh_3_)_3_] and [RuCl_2_(PPh_3_)_2_(dppb)] complexes as precursors. Two of them, characterized as *cis*-[Ru(6m2tu)_2_(PPh_3_)_2_] (**1**) and [Ru(6m2tu)_2_(dppb)] (**2**) (where PPh_3_ = triphenylphosphine; dppb = 1,4-bis(diphenylphosphino)butane; and 6m2tu = 6-methyl-2-thiouracil) (see Fig. 1), have shown potent cytotoxic effect and able to bind DNA^20^. On the other hand, cellular action mechanism of them have not been fully investigated, yet. Therefore, the aim of this study was to evaluate the mechanism underlying of *in vitro* cytotoxicity and *in vivo* action of these ruthenium(II) complexes with 6-methyl-2-thiouracil in human acute promyelocytic leukemia HL-60 cells.

## Material and Methods

### Synthesis of ruthenium(II) complexes with 6-methyl-2-thiouracil

Ruthenium(II) complexes with 6-methyl-2-thiouracil ligand, *cis*-[Ru(6m2tu)_2_(PPh_3_)_2_] (**1**) and [Ru(6m2tu)_2_(dppb)] (**2**), were obtained as previously described by Correa^20^. Briefly, synthesis of **1** and **2** were carried out in a Schlenk reaction flask containing 0.25 mmol of 6-methyl-2-thiouracil ligand dissolved in a methanol (10 mL) solution with 20 μL of triethylamine. After, 0.12 mmol of [RuCl_2_(PPh_3_)_3_] or [RuCl_2_(PPh_3_)(dppb)] precursor was added to the reaction medium. The mixture was maintained under stirring at room temperature for 3 h. Then, the volume was reduced to *c.a*. 2 mL and a yellow solid was formed. The solid was collected by filtration, washed with methanol, diethyl ether and dried under vacuum. All manipulations were performed under argon. All reagents were purchased from Sigma-Aldrich (Sigma-Aldrich Co., Saint Louis, MO, USA) and were used as received.

### *In vitro* assays

#### Cells

HL-60 (human acute promyelocytic leukemia), K-562 (human chronic myelogenous leukemia), HCT116 (human colon carcinoma), HepG2 (human hepatocellular carcinoma), HSC-3 (human oral squamous cell carcinoma), SCC-9 (human oral squamous cell carcinoma), B16-F10 (mouse melanoma), MRC-5 (human lung fibroblast), WT SV40 MEF (wild-type immortalized mouse embryonic fibroblast) and BAD KO SV40 MEF (BAD gene knockout immortalized mouse embryonic fibroblast) cell lines were obtained from American Type Culture Collection (ATCC, Manassas, VA, USA). Human peripheral blood mononuclear cells (PBMC) were isolated using standard Ficoll density gradient from heparinized blood collected from 20- to 35-year-old, non-smoker healthy donors with informed consent (number 031019/2013) approved by Human Ethics Committee of Gonçalo Moniz Institute from Oswaldo Cruz Foundation (IGM-FIOCRUZ/BA), and all experiments were performed in accordance with relevant guidelines and regulations. Cells were cultured as recommended by ATCC guidelines and a mycoplasma stain kit (Sigma-Aldrich) was used to validate the use of cells free from contamination. Cell viability in all experiments was examined using the trypan blue exclusion (TBE) assay. Over 90% of the cells were viable at the beginning of the culture.

#### Cytotoxicity assay

Cytotoxicity was measured using alamar blue assay and was performed following the procedure that was described previously^21,22^. Briefly, cells were inserted in 96-well plates and incubated overnight. Then, the complexes were dissolved in dimethyl sulfoxide (DMSO, LGC Biotechnology, São Paulo, SP, Brazil) and added to each well and incubated for 72 h. Doxorubicin (purity ≥95%, doxorubicin hydrochloride, Laboratory IMA S.A.I.C., Buenos Aires, Argentina) and oxaliplatin (Sigma-Aldrich Co.) were used as positive controls. Before the end of treatment (4 h for cell lines and 24 h for PBMC), 20 μL of a stock solution (0.312 mg/mL) of alamar blue (resazurin, Sigma-Aldrich Co.) were added to each well. Absorbance at 570 nm and 600 nm was measured using SpectraMax 190 Microplate Reader (Molecular Devices, Sunnyvale, CA, USA).

#### Trypan blue exclusion method

The number of viable cells and non-viable (take up trypan blue) were counted by TBE method. Shortly, 90 μL was removed from the cell suspension and 10 μL of trypan blue (0.4%) was added. Cell counting was performed using a light microscope with a neubauer chamber.

#### Intracellular ruthenium quantification

Intracellular ruthenium quantification in HL-60 cells was evaluated by energy dispersive X-ray spectrometer (EDS)^23^. Cells were fixed in sodium cacodylate buffer (0.1 M sodium cacodylate solution pH 7.4, plus 2.5% glutaraldehyde and 2% paraformaldehyde) for at least 2 h. After washing, cells were dehydrated in an acetone series and embedded in polybed epoxy resin (Polysciences; Warrington, PA). Ultrathin sections were examined under a JEM-1230 transmission electron microscope (TEM) integrated with an EDS microanalytics system (JEOL USA, Inc., Peabody, MA, USA).

#### Morphological analysis

To cell morphology evaluation, slides were prepared using cytospin and stained with May-Grunwald-Giemsa. Morphological changes were assessed by light microscopy (Olympus BX41, Tokyo, Japan) using Image-Pro software (Media Cybernetics, Inc. Silver Spring, USA). Light scattering features were determined by flow cytometry. At least 10^4^ events were recorded per sample using a BD LSRFortessa cytometer along with BD FACSDiva Software (BD Biosciences, San Jose, CA, USA) and Flowjo Software 10 (Flowjo LCC, Ashland, OR, USA). Cellular debris was omitted from the analysis.

#### Apoptosis quantification assay

FITC Annexin V Apoptosis Detection Kit I (ID 556547) (BD Biosciences) was used for apoptosis quantification and the analysis was performed according to the manufacturer’s instructions. Shortly, cells were washed twice with saline solution and resuspended in 100 μL of binding buffer plus 5 μL of propidium iodide (PI) and 5 μL of FITC Annexin V. Then, cells were gently mixed by vortexing and incubated for 15 min at room temperature in the dark. Finally, 400 μL of binding buffer was added to each tube, and the cell fluorescence was determined by flow cytometry, as described above. Percentage of viable, early apoptotic, late apoptotic and necrotic cells were measured. Protection assays using a pan-caspase inhibitor (Z-VAD(Ome)-FMK, Cayman Chemical; Ann Arbor, MI, USA), JNK/SAPK inhibitor (SP 600125; Cayman Chemical), p38 MAPK inhibitor (PD 169316; Cayman Chemical) and MEK inhibitor (U-0126; Cayman Chemical), were also evaluated. In these assays, cells were preincubated for 2 h with 50 µM Z-VAD(Ome)-FMK, 5 µM U-0126, 5 µM SP 600125 or 5 µM PD 169316, followed by incubation with 4 µM of complexes **1** and **2** for 24 h. Cells were then analyzed by FITC Annexin V Apoptosis Detection assay as described above.

#### Measurement of mitochondrial transmembrane potential

Mitochondrial transmembrane potential was determined by retention of dye rhodamine 123 as described previously^24^. Briefly, cells were incubated with rhodamine 123 (5 μg/mL, Sigma-Aldrich Co.) at room temperature for 15 min in dark and washed with saline solution. Cells were incubated again in saline solution for more 30 min in dark and cell fluorescence was determined by flow cytometry as described above.

#### Caspase-3, -8 and -9 activation assays

To investigate the activation of caspase-3, -8 and -9, we used caspase-3 colorimetric assay kit (ID K106-100), caspase-8 colorimetric assay kit (ID K113-100) and caspase-9 colorimetric assay kit (ID K119-100) (all from BioVision Inc.; Milpitas, CA, USA), and the analysis were performed according to the manufacturer’s instructions. Enzyme reactions were performed in a 96-well microplate, and to each reaction mixture, 5 μL of cell lysate was added. Total protein quantification was performed in each sample by Bradford assay using bovine serum albumin (BSA) as standard. Absorbance at 405 nm was measured using a SpectraMax 190 Microplate Reader (Molecular Devices).

#### Measurement of cellular reactive oxygen species levels

The levels of intracellular reactive oxygen species (ROS) were measured according to previously described^25^ using 2′,7′-dichlorofluorescin diacetate (DCF-DA, Sigma-Aldrich Co.). Shortly, cells were washed with saline solution and resuspended in saline solution containing 5 μM of DCF-DA for 30 min in dark at room temperature. Finally, cells were washed with saline solution and cell fluorescence was measured by flow cytometry as described above. Protection assay using the antioxidant N-acetyl-L-cysteine (NAC, Sigma-Aldrich Co.) was also evaluated. In brief, cells were preincubated for 1 h with 5 mM of NAC, followed by incubation with 4 µM of complexes **1** and **2** for 24 h. Cells were then analyzed by FITC Annexin V Apoptosis Detection assay as described above.

#### Phospho-specific ELISA

Phosphorylated histone H2AX (S139) (ID DYC2288-2), JNK2 (T183/Y185) (ID DYC2236-2), p38α (T180/Y182) (DYC869B-2) and ERK1 (T202/Y204) (ID DYC1825-2) expressions were quantified in cell lysates using sandwich ELISA kits (all from R&D Systems, Inc. Minneapolis, MN, USA), and the analysis was performed according to the manufacturer’s instructions. Shortly, cells were lysed in a buffer solution containing 100 mM tris, pH 7.4, 150 mM NaCl, 1 mM EGTA, 1 mM EDTA, 1% triton X-100 and 0.5% sodium deoxycholate plus phosphatase inhibitor cocktail, protease inhibitor cocktail and 1 mM PMSF immediately before use (all from Sigma-Aldrich Co.). Total protein quantification was performed in each sample by Pierce Protein Assay (Thermo Fisher Scientific, Waltham, MA, USA) using BSA as standard. Absorbance at 450 nm was measured using a SpectraMax 190 Microplate Reader (Molecular Devices, Sunnyvale, CA, USA).

### *In vivo* assays

#### Animals

Fifty six specific-pathogen-free (SPF) C.B*-*17 severe combined immunodeficient (SCID) mice (females, 23–26 g) were used in this study. The animals were obtained and maintained at animal facilities from Gonçalo Moniz Institute-FIOCRUZ (Salvador, Bahia, Brazil), and housed in cages with free access to food and water, and kept under a 12:12 h light-dark cycle (lights on at 6:00 a.m.). The animals were treated according to ethical principles for animal experimentation of SBCAL (Brazilian Association of Laboratory Animal Science), Brazil. Experimental protocol have been approved (number 06/2015) by Animal Ethics Committee of Gonçalo Moniz Institute-FIOCRUZ (Salvador, Bahia, Brazil).

#### Human myeloid leukemia xenograft model

Human myeloid leukemia xenograft model was carry out as described previously by Rodrigues *et al*.^26^ with minor modifications. HL-60 cells (2.5 × 10^7^ cells/500 µL) were implanted subcutaneously into the left front armpit of the mice. At the beginning of the experiment, mice were randomly divided into four groups: group 1 animals treated with the vehicle 5% DMSO solution (negative control, n = 14); group 2 animals treated with doxorubicin (positive control, 0.1 mg/kg, n = 14); group 3 animals treated with complex **1** at 20 mg/kg (n = 14); and group 4 animals treated with complex **1** at 40 mg/kg (n = 14). When the tumors reached 100 to 200 mm^3^ (22 days after HL-60 cells injection), the animals were treated through the intraperitoneal route (200 µL per animal) once a day for 13 consecutive days. One day after the end of the treatment, the animals were anesthetized, and peripheral blood samples were collected from brachial artery. Animals were euthanized by anesthetic overdose, and tumors were excised and weighed.

#### Toxicological evolution

To assess toxicological aspects, mice were weighed at the beginning and at the end of the experiment as described previously by Rodrigues *et al*.^26^. Animals were observed for signs of abnormalities throughout the study. Hematological analysis was performed using the Advia 60 hematology system (Bayer, Leverkusen, Germany). Livers, kidneys, lungs and hearts were removed, weighed and examined for any signs of macroscopic lesions, color changes and/or hemorrhages. After macroscopic examination, tumors, livers, kidneys, lungs and hearts were fixed in 4% formalin buffer and embedded in paraffin. Tissue sections were stained with hematoxylin/eosin staining, and a pathologist performed the histological analyses under optical microscopy.

### Statistical analysis

Data are presented as mean ± S.E.M. or inhibitory concentration of 50% (IC_50_) values with their respective 95% confidence intervals obtained by nonlinear regression. Analysis of variance (ANOVA) followed by Student–Newman–Keuls test was used to check differences between experimental groups (*p* < 0.05). Statistical analysis was carry out using GraphPad Prism software (Intuitive Software for Science, San Diego, CA, USA).

## Results

### Ruthenium(II) complexes with 6-methyl-2-thiouracil display potent and selective cytotoxicity in myeloid leukemia cell lines

Inhibitory cell growth effect of ruthenium(II) complexes with 6-methyl-2-thiouracil on seven cancer cell lines, HL-60 (human acute promyelocytic leukemia), K-562 (human chronic myelogenous leukemia), HCT116 (human colon carcinoma), HepG2 (human hepatocellular carcinoma), SCC-4 (human oral squamous cell carcinoma), HSC-3 (human oral squamous cell carcinoma) and B16-F10 (mouse melanoma), and two non-cancer cells, MRC-5 (human lung fibroblast) and PBMC (human peripheral blood mononuclear cells), was measured by alamar blue assay after 72 h of treatment.

Both complexes displayed potent cytotoxicity on all cancer cell lines tested. In special, both complexes exhibited potent and selective cytotoxicity in myeloid leukemia cell lines, HL-60 e K-562, which complex **1** was more potent than complex **2** and positive controls doxorubicin and oxaliplatin. IC_50_ values obtained are shown in Table 1. Complex **1** displayed IC_50_ values ranging from 0.01 to 4.37 µM for HL-60 and HSC-3 cell lines, while displayed IC_50_ values of 5.71 and 2.57 µM for non-cancer cells MRC-5 and PBMC, respectively. Complex **2** exhibited IC_50_ values ranging from 0.13 to 2.04 µM for HL-60 and HSC-3 cell lines, and showed IC_50_ values of 3.06 and 1.25 µM for MRC-5 and PBMC, respectively. IC_50_ values of doxorubicin ranged from 0.02 to 1.43 µM for HL-60 and SCC-4, and presented IC_50_ values of 1.51 and 5.17 µM for MRC-5 and PBMC, respectively. IC_50_ values of oxaliplatin ranged from 0.03 to 6.98 µM for B16-F10 and HepG2, and showed IC_50_ values of 1.54 and 14.88 µM for MRC-5 and PBMC, respectively. The ligand 6-methyl-2-thiouracil was also tested and was not cytotoxic to any cells at concentrations tested (IC_50_ > 175.83 μM). Next, selectivity index (SI) was calculated using the following formula: SI = IC_50_ [non-cancer cells]/IC_50_ [cancer cells]. Table 2 shows the SI obtained. Complex **1** displayed SI of 571-fold (HL-60 versus MRC-5), 28.6-fold (K-562 versus MRC-5), 257-fold (HL-60 versus PBMC) and 12.9 (K-562 versus PBMC), while complex **2** exhibited SI of 23.5-, 9-, 9.6- and 3.7-fold, doxorubicin presented SI of 75.5-, 25.2-, 258.5- and 86.2-fold, and oxaliplatin showed SI of 3.9-, 1.7-, 37.2- and 16.5-fold, respectively for the same cell lines comparison.

**Table 1:** Cytotoxic activity of ruthenium(II) complexes with 6-methyl-2-thiouracil.

Cells	IC_50_ and 95% CI (µM)			
	DOX	OXA	(1)	(2)
**Cancer cells**				
HL-60	0.02 0.02–0.06	0.40 0.03–3.75	0.01 0.01–0.52	0.13 0.03–0.61
K-562	0.06 0.04–0.08	0.90 0.08–9.90	0.20 0.10–0.40	0.34 0.17–0.73
HCT116	0.07 0.04–0.12	4.13 2.67–6.42	1.38 0.76–2.53	1.16 0.94–1.47
HepG2	0.02 0.02–0.08	0.96 0.23–3.94	0.95 0.44–2.07	0.83 0.26–2.65
SCC-4	1.43 1.02–2.02	6.98 3.28–14.94	1.63 0.82–3.27	1.21 0.78–1.90
HSC-3	0.25 0.18–0.36	6.80 4.73–9.75	4.37 2.65–7.24	2.04 1.72–2.46
B16-F10	0.02 0.02–0.04	0.03 0.02–0.05	0.83 0.60–1.16	1.04 0.74–1.47
**Non-cancer cells**				
MRC-5	1.51 1.20–1.92	1.54 0.86–2.85	5.71 2.60–12.55	3.06 2.38–4.01
PBMC	5.17 2.56–10.43	14.88 8.91–24.83	2.57 2.07–3.18	1.25 0.67–2.32

Data are presented as IC_50_ values and their respective 95% confidence interval (95% CI) in µM obtained by nonlinear regression from at least three independent experiments performed in duplicate, measured by alamar blue assay after 72 h of treatment. Cancer cells: HL-60 (human acute promyelocytic leukemia); K-562 (human chronic myelogenous leukemia); HCT116 (human colon carcinoma); HepG2 (human hepatocellular carcinoma); SCC-4 (human oral squamous cell carcinoma); HSC-3 (human oral squamous cell carcinoma); and B16-F10 (mouse melanoma). Non-cancer cells: MRC-5 (human lung fibroblast) and PBMC (human peripheral blood mononuclear cells*)*. Doxorubicin (DOX) and oxaliplatin (OXA) were used as positive controls.

**Table 2:** Selectivity index of ruthenium(II) complexes with 6-methyl-2-thiouracil.

Cancer cells	Non-cancer cells							
	MRC-5				PBMC			
	DOX	OXA	(1)	(2)	DOX	OXA	(1)	(2)
HL-60	75.5	3.9	571	23.5	258.5	37.2	257	9.6
K-562	25.2	1.7	28.6	9	86.2	16.5	12.9	3.7
HCT116	21.6	0.4	4.1	2.6	73.9	3.6	1.9	1.1
HepG2	75.5	1.6	6	3.7	258.5	15.5	2.7	1.5
SCC-4	1.1	0.2	3.5	2.5	3.6	2.1	1.6	1
HSC-3	6	0.3	1.3	1.5	20.7	2.2	0.6	0.6
B16-F10	75.5	51.3	6.9	2.9	258.5	496	3.1	1.2

Data are presented the selectivity index (SI) calculated using the following formula: SI = IC_50_[non-cancer cells]/IC_50_[cancer cells]. Cancer cells: HL-60 (human acute promyelocytic leukemia); K-562 (human chronic myelogenous leukemia); HCT116 (human colon carcinoma); HepG2 (human hepatocellular carcinoma); SCC-4 (human oral squamous cell carcinoma); HSC-3 (human oral squamous cell carcinoma); and B16-F10 (mouse melanoma). Non-cancer cells: MRC-5 (human lung fibroblast) and PBMC (human peripheral blood mononuclear cells*)*. Doxorubicin (DOX) and oxaliplatin (OXA) were used as positive controls.

**Figure 2:**
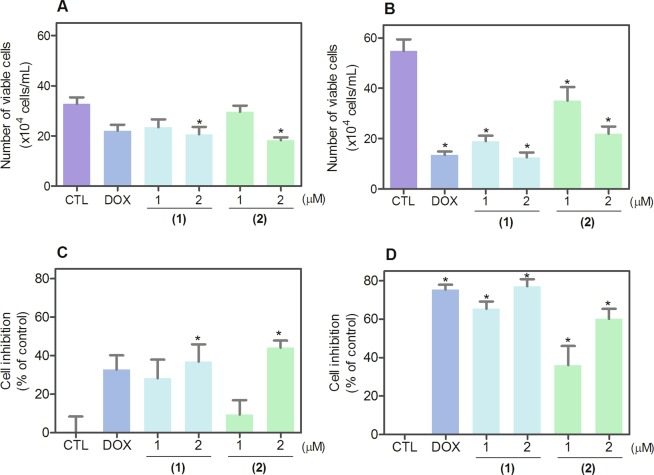
Effect of ruthenium(II) complexes with 6-methyl-2-thiouracil in the cell viability of HL-60 cells determined by trypan blue exclusion assay after 12 (**A**,**C**) and 24 (**B**,**D**) h of treatment. The number of viable cells are presented in (**A**,**B**) and the cell inhibition percentages are presented in (**C**,**D**). Negative control (CTL) was treated with vehicle (0.2% DMSO) used for diluting the complexes, and doxorubicin (DOX, 1 µM) was used as positive control. Data are presented as mean ± S.E.M. of at least three independent experiments performed in duplicate. *p < 0.05 compared with negative control by ANOVA followed by Student Newman-Keuls test.

We confirmed the effect of ruthenium(II) complexes with 6-methyl-2-thiouracil on cell viability and proliferation by trypan blue exclusion assay in HL-60 cells, after 12 and 24 h of treatment (Fig. 2). After 12 h of treatment, complex **1** reduced the number of viable cells by 36.9%, and complex **2** reduced 44.1% at concentration of 2 μM, respectively (no statistically significant reduction was observed with treatment of 1 μM). After 24 h of treatment, complex **1** reduced the number of viable cells by 65.3 and 77.1%, respectively, and complex **2** reduced 36.1 and 60.1% at 1 and 2 μM. None of complexes induced significant (*p* > 0.05) increase in the non-viable cells. Doxorubicin also reduced the number of viable cells after 24 h of treatment.

**Figure 3:**
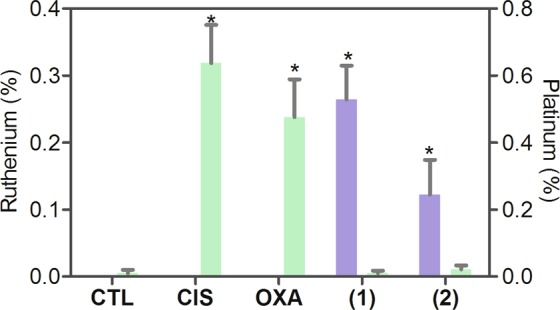
Quantification of intracellular ruthenium in HL-60 cells after 3 h of treatment with 300 µM of ruthenium(II) complexes with 6-methyl-2-thiouracil, as determined by energy dispersive X-ray spectrometer. Untreated cells were used as negative control (CTL). Cisplatin (CIS, 500 µM) and oxaliplatin (OXA, 500 µM) was used as positive controls, and intracellular platinum was determined. Blue bars represent the percent of ruthenium, and green bars represent the percent of platinum. Ten cells were analyzed in each treatment. Data are presented as mean ± S.E.M. *P < 0.05 compared with negative control by ANOVA, followed by Student-Newman-Keuls test.

Intracellular quantification of ruthenium was assessed with an energy dispersive X-ray spectrometer in HL-60 cells treated with ruthenium(II) complexes with 6-methyl-2-thiouracil after 3 h of incubation (Fig. 3). Cisplatin and oxaliplatin were used as positive controls, and intracellular quantification of platinum was also measured. We were able to detect ruthenium in HL-60 cells treated with both complexes, as well as, we were able to quantify platinum in HL-60 cells treated with cisplatin and oxaliplatin.

### Ruthenium(II) complexes with 6-methyl-2-thiouracil trigger caspase-mediated apoptosis in HL-60 cells

**Figure 4:**
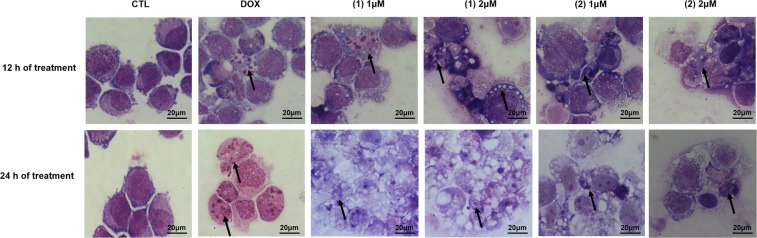
Effect of ruthenium(II) complexes with 6-methyl-2-thiouracil in the morphological analysis of HL-60 cells, assessed by May-Grunwald-Giemsa staining and examined by light microscopy (bar = 20 µm), after 12 and 24 h of treatment. Negative control (CTL) was treated with vehicle (0.2% DMSO) used for diluting the complexes, and doxorubicin (DOX, 1 µM) was used as positive control. Arrows indicated cells with reduction in the cell volume, cell vascularization or fragmented DNA.

**Figure 5:**
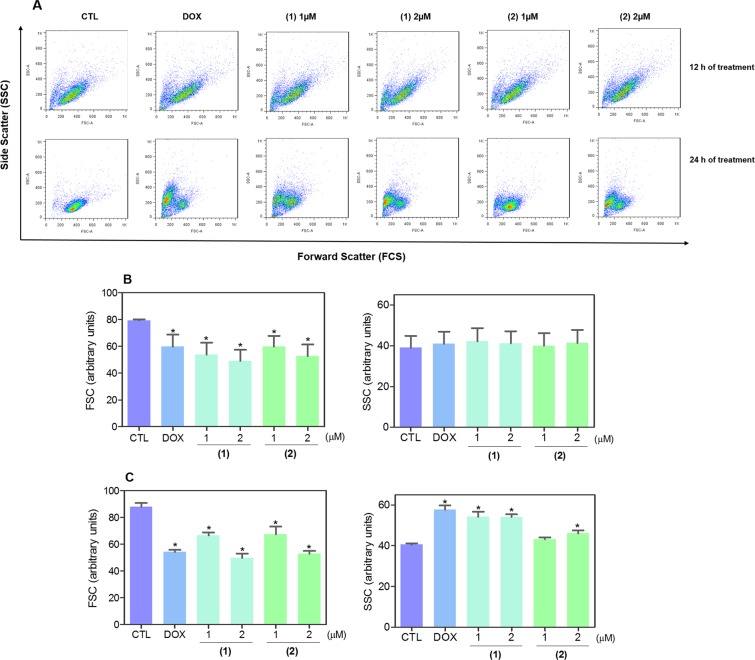
Effect of ruthenium(II) complexes with 6-methyl-2-thiouracil in the morphological analysis of HL-60 cells, assessed by light scattering features using flow cytometry. Representative flow cytometry dot plots are presented in (**A**) and quantification of forward light scatter (FSC) and side-scattered light (SSC) after 12 h of treatment are presented in (**B**) and after 24 h of treatment in (**C**). FSC and SSC were measured as parameters of cell-surface area or size and cell granularity or internal complexity, respectively. Negative control (CTL) was treated with vehicle (0.2% DMSO) used for diluting the complexes, and doxorubicin (DOX, 1 µM) were used as positive control. Data are presented as mean ± S.E.M. of at least three independent experiments performed in duplicate. Ten thousand events were evaluated per experiment, and cellular debris was omitted from analysis. *p < 0.05 compared with the negative control by ANOVA followed by Student Newman-Keuls test.

Using a light microscope, we analyzed the effect of ruthenium(II) complexes with 6-methyl-2-thiouracil in the cell morphology of HL-60 cells stained with May-Grunwald-Giemsa. Both complexes caused reduction in the cell volume, vacuolization, chromatin condensation and DNA fragmentation were observed after treatment with both complexes (Fig. 4). Moreover, we also found cell shrinkage, as observed by the decrease in forward light scattering (FSC), and nuclear condensation, as observed by an increase in lateral dispersion (SSC), in HL-60 cells treated with both complexes (Fig. 5). These alterations are consistent with apoptotic cell death. The treatment with doxorubicin also presented changes associated with apoptosis.

**Figure 6:**
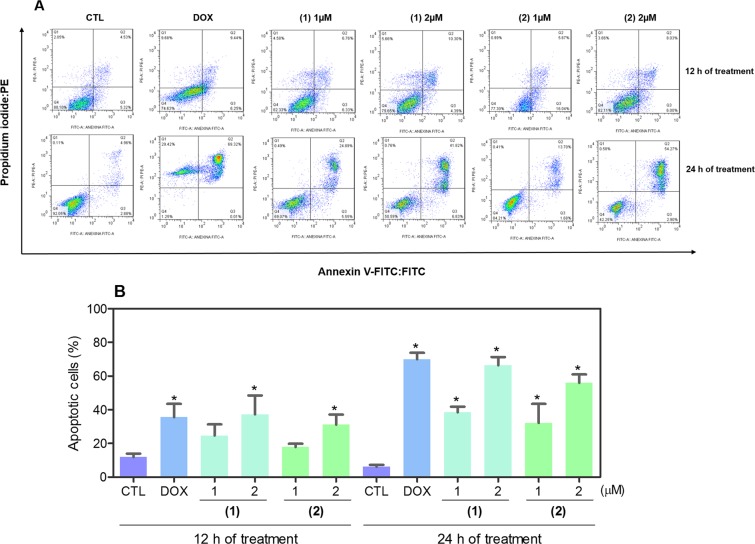
Effect of ruthenium(II) complexes with 6-methyl-2-thiouracil in the induction of apoptosis in HL-60 cells determined by flow cytometry using annexin V-FITC/PI staining after 12 and 24 h of treatment. (**A**) Representative flow cytometric dot plots showing the percentage of cells in viable (annexin V-FITC negative and PI negative cells), early apoptotic (annexin V-FITC positive, but PI negative cells), late apoptotic (annexin V-FITC positive and PI positive cells) and necrotic stages (PI positive, but annexin V-FITC negative cells). (**B**) Quantification of apoptotic HL-60 cells (annexin V-FITC positive cells). Negative control (CTL) was treated with vehicle (0.2% DMSO) used for diluting the complexes, and doxorubicin (DOX, 1 µM) was used as positive control. Data are presented as mean ± S.E.M. of at least three independent experiments performed in duplicate. Ten thousand events were evaluated per experiment, and cellular debris was omitted from analysis. *p < 0.05 compared with negative control by ANOVA followed by Student Newman-Keuls test.

To confirm the apoptosis induction by ruthenium(II) complexes with 6-methyl-2-thiouracil in HL-60 cells, annexin V/propidium iodide double staining was performed to measure phosphatidylserine exposure and loss of membrane integrity, respectively, and the numbers of viable, early apoptotic, late apoptotic and necrotic cells were quantified. Both complexes strongly increased apoptotic cells after 12 and 24 h of treatment (Fig. 6). After 12 h of treatment, complex **1** led 37.2% of apoptosis, while complex **2** caused 31.1% at concentration of 2 μM, respectively (no statistically significant apoptosis induction was observed with the treatment of 1 μM). After 24 h of treatment, complex **1** led 38.5 and 66.4% of apoptosis at concentrations of 1 and 2 μM, respectively, and complex **2** caused 32.1 and 55.9%. Doxorubicin also induced apoptosis in HL-60 cells.

**Figure 7:**
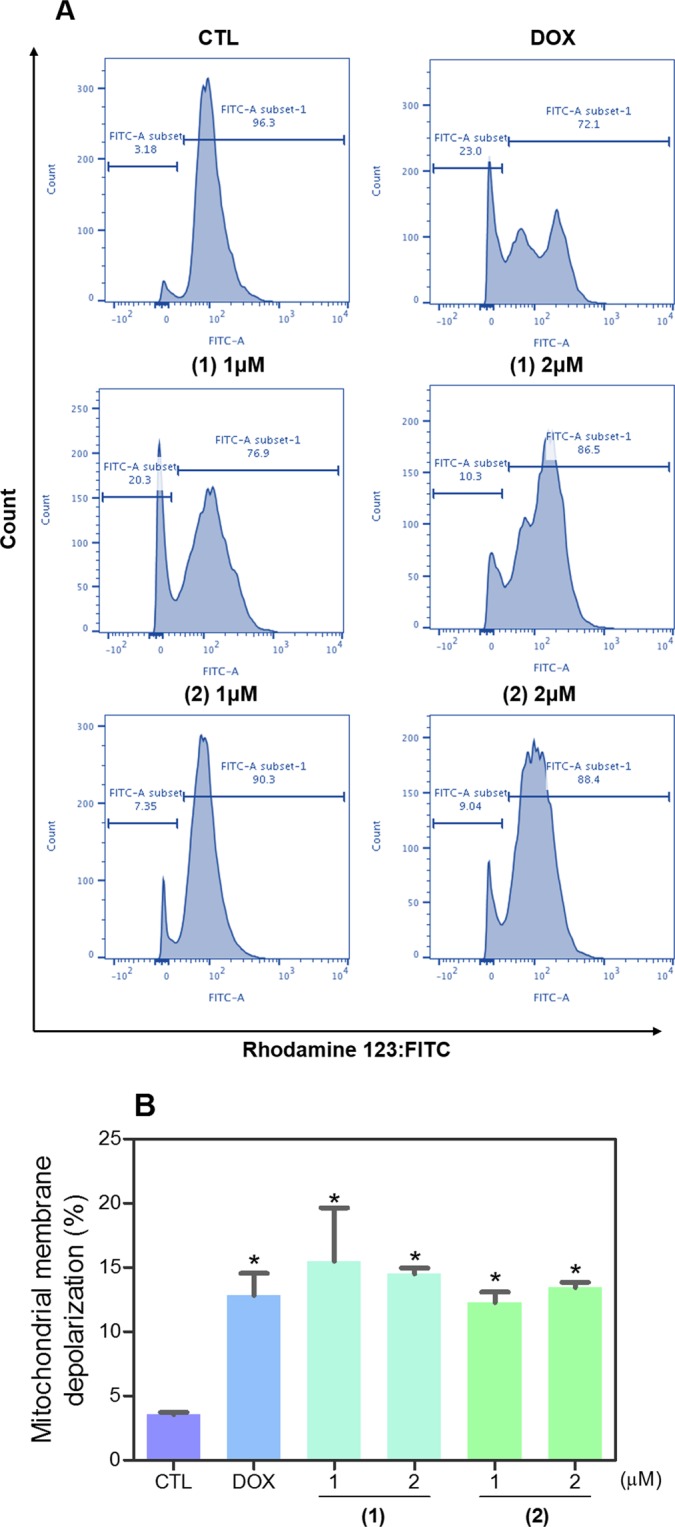
Effect of ruthenium(II) complexes with 6-methyl-2-thiouracil in mitochondrial membrane potential in HL-60 cells after 24 h of treatment determined by flow cytometry using rhodamine 123 staining. (**A**) Representative flow cytometric histograms. (**B**) Quantification of the percentage of mitochondrial membrane potential. Negative control (CTL) was treated with vehicle (0.2% DMSO) used for diluting the complexes, and doxorubicin (DOX, 1 µM) were used as positive control. Data are presented as mean ± S.E.M. of at least three independent experiments performed in duplicate. Ten thousand events were evaluated per experiment, and cellular debris was omitted from analysis. *p < 0.05 compared with negative control by ANOVA followed by Student Newman-Keuls test.

**Figure 8:**
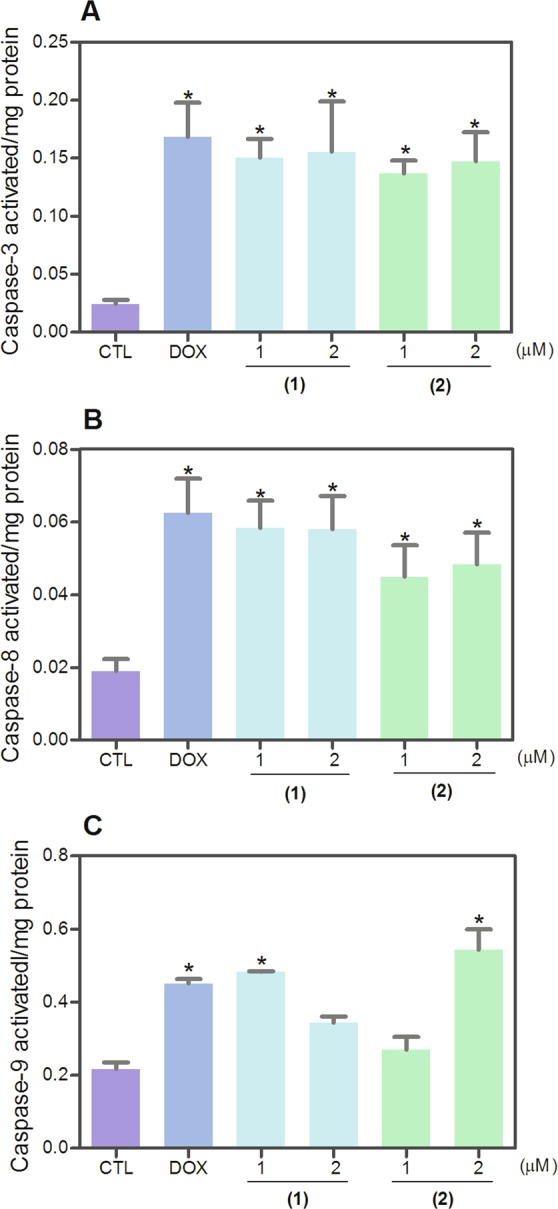
Effect of ruthenium(II) complexes with 6-methyl-2-thiouracil in caspase-3 (**A**), -8 (**B**) and -9 (**C**) activities in HL-60 cells determined by colorimetric assay after 24 h of treatment. Negative control (CTL) was treated with vehicle (0.2% DMSO) used for diluting the complexes, and doxorubicin (DOX, 1 µM) were used as positive control. Data are presented as mean ± S.E.M. of at least three independent experiments performed in duplicate. *p < 0.05 compared with negative control by ANOVA followed by Student Newman-Keuls test.

**Figure 9:**
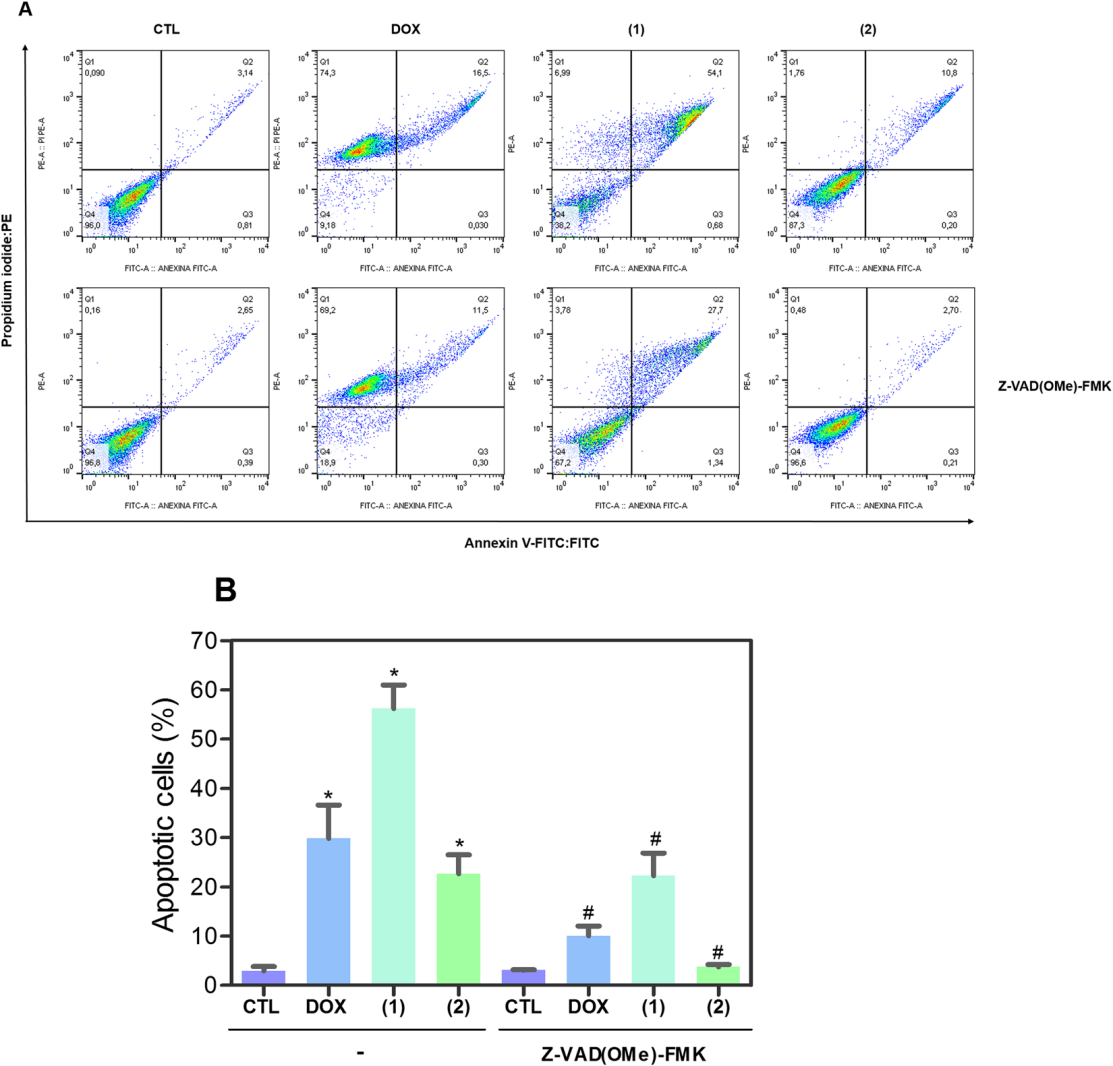
Effect of a pan-caspase inhibitor (Z-VAD(OMe)-FMK) in the apoptosis induced by ruthenium(II) complexes with 6-methyl-2-thiouracil in HL-60 cells determined by flow cytometry using annexin V-FITC/PI staining. (**A**) Representative flow cytometric dot plots showing the percentage of cells in viable (annexin V-FITC negative and PI negative cells), early apoptotic (annexin V-FITC positive, but PI negative cells), late apoptotic (annexin V-FITC positive and PI positive cells) and necrotic stages (PI positive, but annexin V-FITC negative cells). (**B**) Quantification of apoptotic HL-60 cells (annexin V-FITC positive cells). Cells were pre-treated for 2 h with 50 µM Z-VAD(OMe)-FMK, then incubated with the complexes at 2 μM for 24 h. Negative control (CTL) was treated with vehicle (0.2% DMSO) used for diluting the complexes, and doxorubicin (DOX, 1 µM) was used as positive control. Data are presented as mean ± S.E.M. of at least three independent experiments performed in duplicate. Ten thousand events were evaluated per experiment, and cellular debris was omitted from analysis. *p < 0.05 compared with negative control by ANOVA followed by Student Newman-Keuls test. ^#^*p* < 0.05 com*p*ared with respective treatment without inhibitor by ANOVA followed by Student Newman-Keuls test.

Mitochondrial transmembrane potential was also examined in HL-60 cells treated with ruthenium(II) complexes with 6-methyl-2-thiouracil using the retention of dye rhodamine 123 assay by flow cytometry. Both complexes caused loss of the mitochondrial transmembrane potential (Fig. 7). Next, activation of the effector (caspase-3) and initiator (caspases-8 and -9) caspases was also studied. Both complexes induced the activation of all caspases analyzed (Fig. 8). In addition, cotreatment with a pan-caspase inhibitor (Z-VAD(OMe)-FMK) reduced the apoptosis caused by both complexes, indicating a caspase-mediated apoptotic cell death. (Fig. 9). Doxorubicin also induced depolarization of mitochondrial transmembrane potential and led to apoptosis through caspases pathways in HL-60 cells.

Finally, viability of BAD (Bcl-2-associated death promoter) mutant cell line BAD KO SV40 MEF (immortalized mouse embryonic fibroblast with the BAD gene knocked out) and its parental cell line WT SV40 MEF (wild-type immortalized mouse embryonic fibroblasts) were examined after 72 h of treatment with ruthenium(II) complexes with 6-methyl-2-thiouracil by alamar blue assay to assess the role of BAD protein in cytotoxicity caused by these complexes. BAD is an important pro-apoptotic protein belong to Bcl-2 family that is involved in early stages of apoptosis. IC_50_ values for complex **1** were 3.1 μM for BAD KO SV40 MEF cell line and 3.5 μM for WT SV40 MEF cell line, while complex **2** showed IC_50_ values of 4.2 μM for BAD KO SV40 MEF cell line and 3.9 μM for WT SV40 MEF cell line, suggesting that BAD gene is not essential for their cytotoxicity. Doxorubicin presented IC_50_ values of 0.04 and 0.41 μM in WT SV40 MEF and BAD KO SV40 MEF cell lines, respectively.

The effect of ruthenium(II) complexes with 6-methyl-2-thiouracil on ROS levels was also evaluated in HL-60 cells. However, the complexes did not increase significantly ROS levels after 1 or 3 h of incubation (data not shown). In addition, cotreatment with the antioxidant NAC did not reduce the apoptosis induced by complexes (data not shown).

### Ruthenium(II) complexes with 6-methyl-2-thiouracil cause DNA double-strand break and apoptosis through JNK/p38 pathways in HL-60 cells

**Figure 10:**
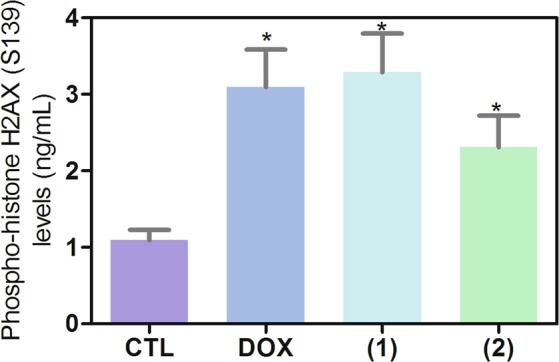
Effect of ruthenium(II) complexes with 6-methyl-2-thiouracil in phospho-histone H2AX (S139) expression, as determined by phospho-specific ELISA in HL-60 cells treated with the complexes at 2 µM for 24 h incubation. Negative control (CTL) was treated with vehicle (0.2% DMSO) used for diluting the complexes, and doxorubicin (DOX, 1 µM) was used as positive control. Data are presented as mean ± S.E.M. of at least three independent experiments performed in duplicate. *p < 0.05 compared with negative control by ANOVA followed by Student Newman-Keuls test.

**Figure 11:**
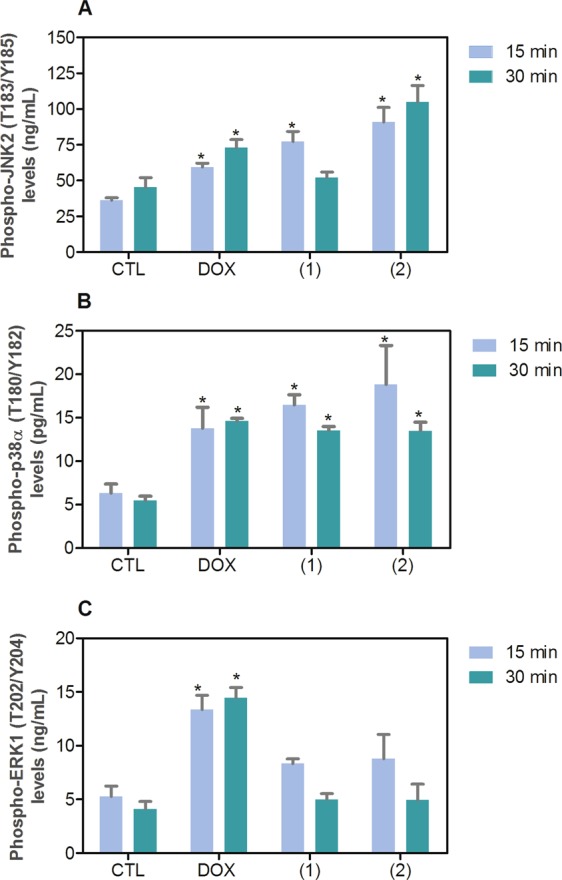
Effect of ruthenium(II) complexes with 6-methyl-2-thiouracil in phospho-JNK2 (T183/Y185), phospho-p38α (T180/Y182) and phospho-ERK1 (T202/Y204) expressions determined by phospho-specific ELISA in HL-60 cells treated with the complexes at 2 µM for 15 and 30 min incubation. (**A**) Quantification of phospho-JNK2 (T183/Y185) expression. (**B**) Quantification of phospho-p38α (T180/Y182) expression. (**C**) Quantification of phospho-ERK1 (T202/Y204) expression. Negative control (CTL) was treated with vehicle (0.2% DMSO) used for diluting the complexes, and doxorubicin (DOX, 1 µM) was used as positive control. Data are presented as mean ± S.E.M. of at least three independent experiments performed in duplicate. *p < 0.05 compared with negative control by ANOVA followed by Student Newman-Keuls test.

**Figure 12:**
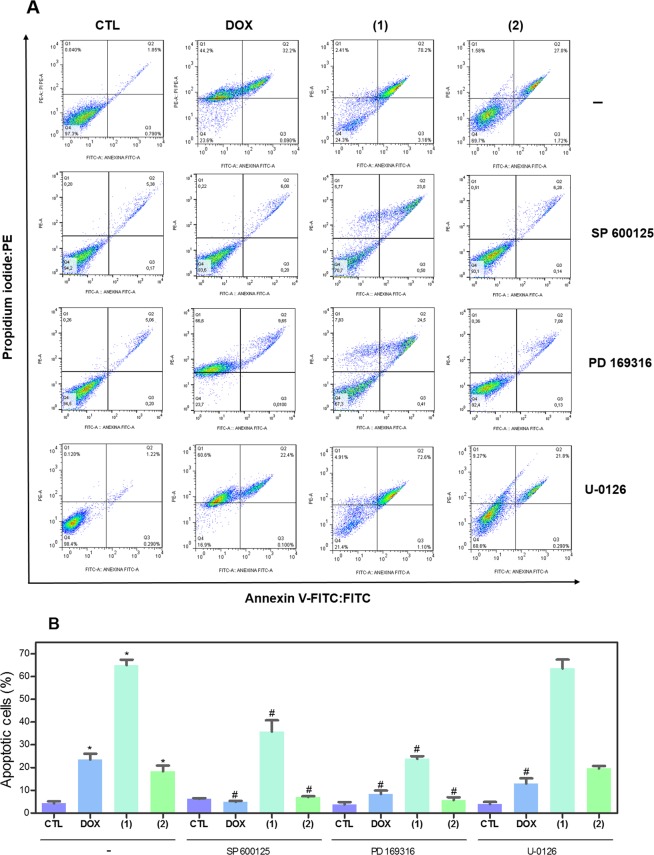
Effect of JNK/SAPK inhibitor (SP 600125), p38 MAPK inhibitor (PD 169316) and MEK inhibitor (U-0126) on the apoptosis induced by ruthenium(II) complexes with 6-methyl-2-thiouracil in HL-60 cells, as determined by flow cytometry using Annexin V-FITC/PI staining. (**A**) Representative flow cytometric dot plots showing the percentage of cells in viable (annexin V-FITC negative and PI negative cells), early apoptotic (annexin V-FITC positive, but PI negative cells), late apoptotic (annexin V-FITC positive and PI positive cells) and necrotic stages (PI positive, but annexin V-FITC negative cells). (**B**) Quantification of apoptotic HL-60 cells (annexin V-FITC positive cells). For protection assays, cells were pretreated for 2 h with 5 µM U-0126, 5 µM SP 600125 or 5 µM PD 169316 and then incubated with the complexes at 2 µM for 24 h. Negative control (CTL) was treated with vehicle (0.2% DMSO) used for diluting the complexes, and doxorubicin (DOX, 1 µM) was used as positive control. Data are presented as mean ± S.E.M. of at least three independent experiments performed in duplicate. Ten thousand events were evaluated per experiment, and cellular debris was omitted from analysis. *P < 0.05 compared with negative control by ANOVA, followed by Student-Newman-Keuls test. ^#^*P* < 0.05 compared with respective treatment without inhibitor by ANOVA, followed by Student-Newman-Keuls test.

Both DNA damage and mitogen-activated protein kinase (MAPK) signaling are involved in cell death caused by many xenobiotics including some antineoplastic drugs. Therefore, we decided study the role of DNA damage and MAPK pathway in the apoptosis induced by ruthenium(II) complexes with 6-methyl-2-thiouracil in HL-60 cells. To assess DNA double-strand break, phospho-histone H2AX (S139) expression was quantified after 24 h of incubation. In addition, complexes-induced apoptosis was measured in HL-60 cells cotreated with MAPK inhibitors. Phospho-JNK2 (T183/Y185), phospho-p38α (T180/Y182) and phospho-ERK1 (T202/Y204) expressions were also quantified after 15 and 30 min of incubation. Treatment with both complexes caused augment of phosphorylation of histone H2AX (S139) (Fig. 10), JNK2 (T183/Y185) (Fig. 11A) and phospho-p38α (T180/Y182) (Fig. 11B), but not phospho-ERK1 (T202/Y204) (Fig. 11C). Moreover, cotreatment with a JNK/SAPK inhibitor (SP 600125) and a p38 MAPK inhibitor (PD 169316), but not a MEK (mitogen-activated protein kinase kinase) inhibitor (U-0126) that inhibits the activation of ERK1/2, reduced the apoptosis caused by both complexes, indicating apoptotic cell death through JNK/p38 pathways in HL-60 cells (Fig. 12).

### Ruthenium(II) complex with 6-methyl-2-thiouracil reduces HL-60 cell growth in xenograft model

**Figure 13:**
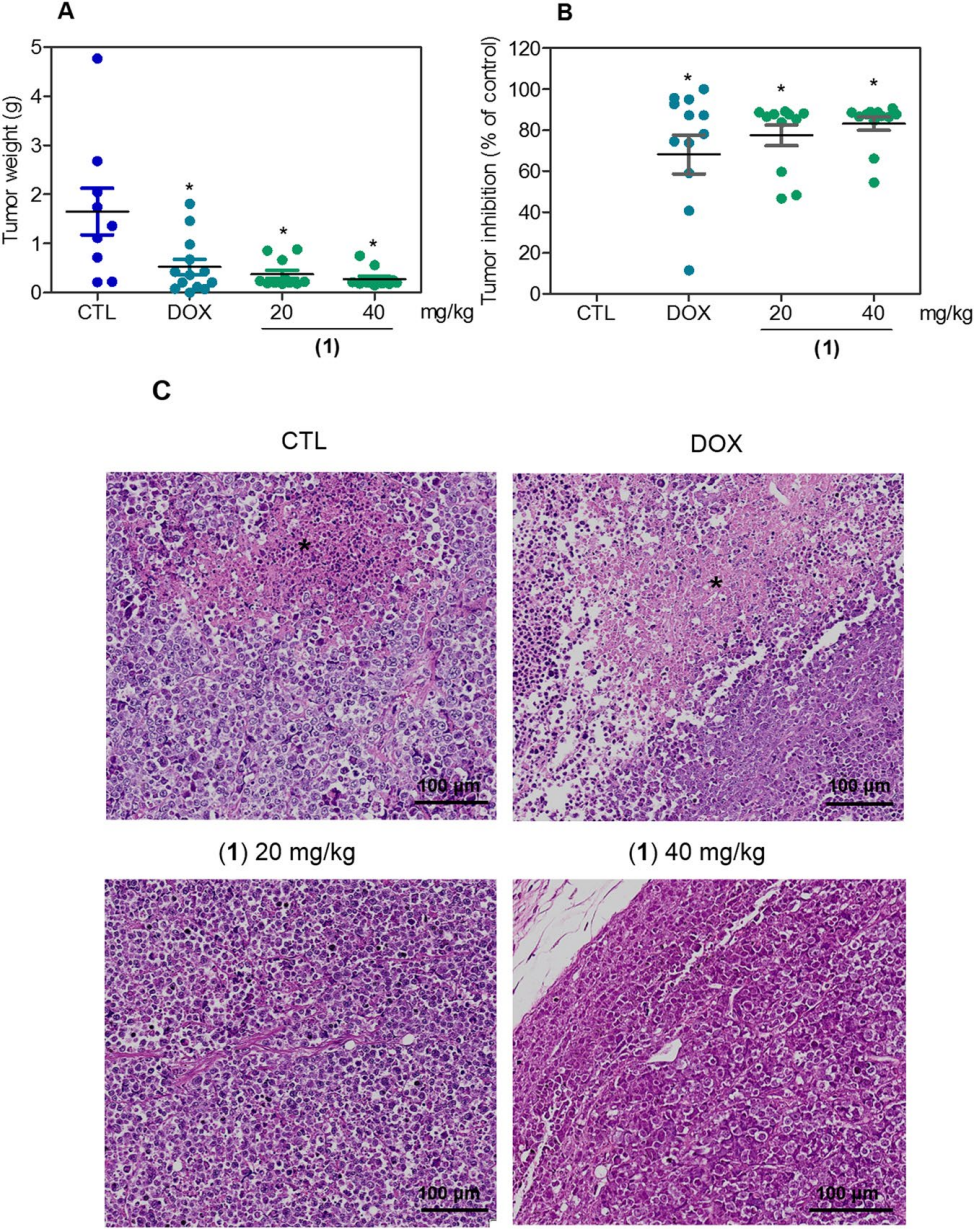
*In vivo* antitumor activity of ruthenium(II) complexes with 6-methyl-2-thiouracil in C.B-17 SCID mice with HL-60 cell xenografts. (**A**) Tumor weight (g) after treatment. (**B**) Tumor inhibition (% of control) after treatment. Data are presented as mean ± S.E.M. of 14 animals. *P < 0.05 compared with negative control by ANOVA, followed by Student-Newman-Keuls test. (**C**) Representative histological analysis of tumors stained with hematoxylin and eosin and analyzed by optical microscopy. Asterisks represent areas with tumor necrosis. When the tumors reached 100 to 200 mm^3^, the animals were treated through the intraperitoneal route for 13 consecutive days. Negative control (CTL) was treated with vehicle (5% DMSO) used for diluting the complexes, and doxorubicin (DOX, 0.1 mg/kg) was used as positive control.

*In vivo* anti-leukemia activity of ruthenium(II) complex with 6-methyl-2-thiouracil was studied in C.B-17 SCID mice engrafted with HL-60 cells. Since complex **1** was more potent than complex **2**, only complex **1** was used in the *in vivo* anti-leukemia model. When the tumors reached 100 to 200 mm^3^ (22 days after HL-60 cells injection), the animals were treated with complex **1** at doses of 20 and 40 mg/kg by intraperitoneal injections once a day for 13 consecutive days. Both doses were able to inhibit HL-60 cell development in mice (Fig. 13A,B). In the end of the treatment, the mean of tumor mass weight of the negative control animals was 1.4 ± 0.4 g. In the group of the animals treated with lower and higher doses of complex **1**, the mean of tumor mass weights was 0.4 ± 0.1 and 0.3 ± 0.1 g, respectively. Tumor mass inhibition rate were 73.1 and 79.9%, respectively. Doxorubicin, at dose of 0.1 mg/kg, reduced tumor weight by 62.0%. In the histomorphological analyses, tumors exhibited malignant cells of abundant and granular cytoplasm, with 2-or more distinct nucleoli with a predominant myeloid morphology. These cells were often arranged in agglomerates with sparse/or without extracellular matrix, mostly in negative control and doxorubicin groups. For the groups treated with complex **1** (20 and 40 mg), areas with nodular/encapsulated growth and extracellular matrix formation were more evident, but multifocal areas of necrosis was more frequent in the negative control and doxorubicin groups (Fig. 13B).

Body weight of the animals at the beginning and at the end of the experiment, hematological analysis of peripheral blood, wet weight of liver, kidney, lung and heart, and their histological analysis, were performed to evaluate toxicology characteristics of ruthenium(II) complex with 6-methyl-2-thiouracil treatment in mice. Only doxorubicin treatment decreased body weight of C.B-17 SCID mice bearing HL-60 cells (*P* < 0.05). No significant chances were found in liver, kidney, lung or heart weight of none group (*P* > 0.05) (data not shown). Moreover, no alterations were found in hematological parameters of peripheral blood of any group (*P* > 0.05) (data not shown).

Morphological analyses of liver, kidneys, lungs and hearts in all groups were performed. In the livers, acinar architecture and centrilobular vein were also preserved in all groups. Focal areas of inflammation and coagulation necrosis were observed in all experimental groups. Additionally, focal areas of steatosis were observed in the negative control and complex **1** (20 and 40 mg/kg) groups. Other findings, such as congestion and hydropic degeneration were found in all groups, ranging from mild to moderate. In the lungs, architecture of the parenchyma was partially maintained in all groups, observing a thickening of the alveolar septum with decreased airspace, ranging from mild to moderate. Histopathological analyses of lungs revealed significant inflammation predominantly of mononuclear cells, edema, congestion and hemorrhage, ranging mild to severe. It is important to note that the inflammation was more evident in animals treated with complex **1** (20 and 40 mg/kg). In addition, tumor nodules were observed only in one animal treated with doxorubicin. In the kidneys, tissue architecture was preserved in all experimental groups. Histopathological changes included vascular congestion and thickening of basal membrane of renal glomerulus with decreased urinary space were observed in all kidneys, ranging from mild to moderate. Coagulation necrosis was observed in renal tubules in groups treated with doxorubicin and complex **1** (20 and 40 mg/kg). Histopathological analysis of animal hearts did not show alterations in any group.

## Discussion

Herein, we reported for the first time *in vitro* cellular underlying mechanism and *in vivo* effectiveness of the ruthenium(II) complexes with 6-methyl-2-thiouracil *cis*-[Ru(6m2tu)_2_(PPh_3_)_2_] and [Ru(6m2tu)_2_(dppb)] in HL-60 cells. Gold(I), palladium(II) and ruthenium(II) complexes containing 2-thiouracil derivatives have been previously evaluated as potential antineoplastic agents; however, these complexes were only preliminarily investigated on cell-based assays^9,17,18^.

Aminophosphine-thiolate gold(I) complex with 2-thiouracil induced cytotoxicity to HeLa (human cervical cancinoma) and MCF-7 (human breast adenocarcinoma) cell lines, and showed a potent inhibition of thioredoxin reductase activity^18^. Some palladium(II) complexes with 2-thiouracil ligands caused cytotoxicity to A-498 (human kidney carcinoma), MCF-7, Evsa-T (human breast carcinoma), H226 (human lung squamous cell carcinoma), IGROV (human ovarian carcinoma), M19-MEL (human melanoma) and WiDr (human colon carcinoma)^17^. Herein, both ruthenium(II) complexes with 6-methyl-2-thiouracil tested exhibited potent and selective cytotoxic effect in myeloid leukemia cells, and complex **1** was more potent than complex **2**, doxorubicin and oxaliplatin. These data corroborate with our previous work, where these complexes were evaluated in a small panel of cancer cells (B16-F10, HepG2, K-562, and HL-60)^20^.

Both complexes were detected into the cells, reduced cell proliferation, increased phosphatidylserine externalization, caspase-3, -8 and -9 activation and loss of mitochondrial transmembrane potential in HL-60 cells. Cotreatment with Z-VAD(OMe)-FMK, a pan-caspase inhibitor, reduced complexes-induced apoptosis. Additionally, the complexes induced phosphorylation of histone H2AX (S139), which monitor the DNA double-strand break^27^, JNK2 (T183/Y185) and p38α (T180/Y182), and cotreatment with JNK/SAPK inhibitor and p38 MAPK inhibitor, but not ERK1/2, partially prevented complexes-induced apoptosis, indicating activation of apoptosis through JNK/p38 pathways, indicating that ruthenium(II) complexes with 6-methyl-2-thiouracil cause DNA double-strand break and trigger caspase-mediated apoptosis through JNK/p38 pathways in HL-60 cells.

JNK/SAPK (isoforms JNK-1, JNK-2 and JNK-3), p38 MAPK (isoforms p38α, p38β, p38γ and p38δ) and ERK1/2 pathways belong to the MAPK family and are involved in different cellular responses, including both cancer cell proliferation and cell death. Interestingly, DNA damage-induced cell death have been involved with activation of JNK and p38 MAPK by expression of pro-apoptotic factors^28^. In fact, we demonstrated that ruthenium(II) complexes with 6-methyl-2-thiouracil induced DNA double-strand break and trigger caspase-mediated apoptosis through JNK/p38 pathways in HL-60 cells. On the other hand, ERK1/2 pathway may active both pro-survival and pro-apoptotic factors. During DNA damage stimuli, e.g. exposition to platinum complexes and ionizing radiation, activation of ERK1/2 pathway causes apoptosis^29–31^. Herein, we demonstrated that ERK1/2 pathway is not essential to the apoptosis induced by ruthenium(II) complexes with 6-methyl-2-thiouracil in HL-60 cells.

In recent studies, ruthenium(II) complex with methylimidazole induced cell cycle arrest at G_0_/G_1_ phase and caused apoptosis through ROS, MAPK and AKT signaling pathways in human lung carcinoma A549 cells^32^, meanwhile ruthenium(II) complex with xanthoxylin caused S-phase arrest and ERK1/2-mediated apoptosis in HepG2 cells by a p53-independent pathway^10^. Similar results were found by Neves and collaborators^16^ with ruthenium complexes containing heterocyclic thioamidates that caused caspase-mediated apoptosis through MAPK signaling in HepG2 cells. Ruthenium(II) complexes with piplartine induced MAPK- and p53-dependent apoptosis in HCT116 cells by a ROS-mediated pathway^7,15^. Apoptosis in A549 cells by mitochondrial homeostasis destruction and death receptor signaling pathways can be also induced by ruthenium(II) polypyridyl complex^33^. Mazuryk and collaborators^34^ revealed that ruthenium(II) complexes with nitroimidazole derivatives of polypyridyl caused caspase-independent cell death by ROS formation, including hydrogen peroxide and peroxyl radicals, and intracellular Ca^2+^ homeostasis disruption in human pancreas carcinoma PANC-1 cells. Ruthenium(II) complexes containing 5-fluorouracil^11^ and thymine^12^ also led caspase-mediated apoptosis in HCT116 and HL-60 cells, respectively.

Complex **1** also inhibited HL-60 cell growth in xenograft model. In mice bearing A549 xenografts, ruthenium(II) imidazole complex also reduced the cancer cell growth^35^. Combination of ruthenium(II)-arene complex and erlotinib inhibited *in vivo* A2780 cell (human ovarian carcinoma) development in a xenograft tumor model^36^. Ruthenium complex with phenylterpyridine derivative inhibited *in vivo* A375 (human skin melanoma) cell development in a xenograft tumor model^37^. Moreover, ruthenium(II) complex with xanthoxylin also presented *in vivo* antitumor effect in C.B-17 SCID mice engrafted with HepG2 cells^10^.

## Conclusion

**Figure 14:**
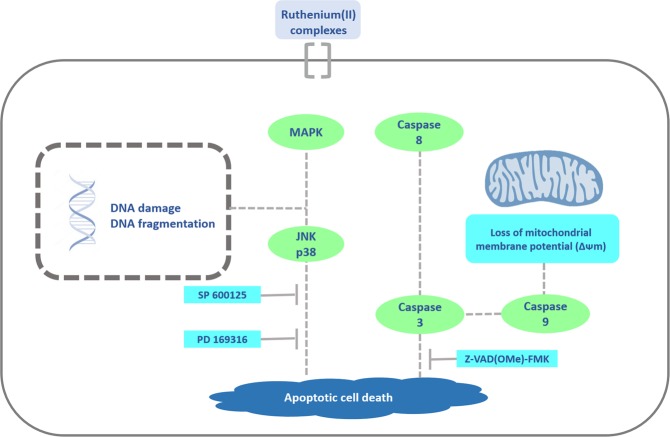
Summary of the mechanisms of action of ruthenium(II) complexes with 6-methyl-2-thiouracil in HL-60 cells.

In conclusion, ruthenium(II) complexes **1** and **2** display potent and selective cytotoxicity in myeloid leukemia cell lines, and can be detected into HL-60 cells. In studies of *in vitro* cellular underlying mechanism, the complexes cause DNA double-strand break and trigger caspase-mediated apoptosis through JNK/p38 pathways in HL-60 cells (Fig. 14). Finally, complex **1** also reduced HL-60 cell growth in xenograft model, indicating that ruthenium(II) complexes with 6-methyl-2-thiouracil are novel promising antileukemic drug candidates.
